# Selective Upregulation of CTLA-4 on CD8^+^ T Cells Restricted by HLA-B*35Px Renders them to an Exhausted Phenotype in HIV-1 infection

**DOI:** 10.1371/journal.ppat.1008696

**Published:** 2020-08-06

**Authors:** Shokrollah Elahi, Shima Shahbaz, Stan Houston

**Affiliations:** 1 School of Dentistry, Faculty of Medicine and Dentistry, University of Alberta, Edmonton, Canada; 2 Department of Oncology, Cross Cancer Institute, University of Alberta, Edmonton, Canada; 3 Department of Medical Microbiology and Immunology, Faculty of Medicine and Dentistry, University of Alberta, Edmonton, Canada; 4 Li Ka Shing Institute of Virology, University of Alberta, Edmonton, Canada; 5 Department of Medicine, Faculty of Medicine and Dentistry, University of Alberta, Edmonton, Canada; Vaccine Research Center, UNITED STATES

## Abstract

HLA-B*35Px is associated with HIV-1 disease rapid progression to AIDS. However, the mechanism(s) underlying this deleterious effect of this HLA allele on HIV-1 infection outcome has not fully understood. CD8^+^ T cells play a crucial role to control the viral replication but impaired CD8^+^ T cells represent a major hallmark of HIV-1 infection. Here, we examined the effector functions of CD8^+^ T cells restricted by HLA-B*35Px (HLA-B*35:03 and HLA-B*35:02), HLA-B*27/B57 and non-HLA-B*27/B57 (e.g. HLA-A*01, A*02, A*03, A*11, A*24, A*26, B*40, B*08, B*38, B*44). CD8^+^ T cells restricted by HLA-B*35Px exhibited an impaired phenotype compared with those restricted by HLA-B*27/B57 and even non-HLA-B*27/B57. CD8^+^ T cells restricted by non-HLA-B*27/B57 when encountered their cognate epitopes upregulated TIM-3 and thus became suppressed by regulatory T cells (Tregs) via TIM-3: Galectin-9 (Gal-9). Strikingly, CD8^+^ T cells restricted by HLA-B*35Px expressed fewer TIM-3 and therefore did not get suppressed by Tregs, which was similar to CD8^+^ T cells restricted by HLA-B*27/B57. Instead, CD8^+^ T cells restricted by HLA-B*35Px upon recognition of their cognate epitopes upregulated CTLA-4. The transcriptional and impaired phenotype (e.g. poor effector functions) of HIV-specific CD8^+^ T cells restricted by HLA-B*35 was related to persistent CTLA-4, elevated Eomes and blimp-1 but poor T-bet expression. As such, anti-CTLA-4 antibody, Ipilimumab, reversed the impaired proliferative capacity of antigen-specific CD8^+^ T cells restricted by HLA-B*35Px but not others. This study supports the concept that CD8^+^ T resistance to Tregs-mediated suppression is related to allele restriction rather than the epitope specificity. Our results aid to explain a novel mechanism for the inability of HIV-specific CD8^+^ T cells restricted by HLA-B*35Px to control viral replication.

## Author summary

A rare group of HIV-infected individuals with HLA-B*35Px rapidly progress to AIDS but those with HLA-B*27 and HLA-B*57 spare disease progression. Previous studies have suggested that viral mutation may prevent a robust immune response against the virus in these with HLA-B*35Px. However, the functionality of HIV-specific CD8^+^ T cells restricted by HLA-B*35Px remains unclear. In this study, we demonstrate that HIV-specific CD8^+^ T cells restricted by HLA-B*35Px (HLA-B*35:03 and HLA-B*35:02) exhibit an impaired phenotype (e.g. low proliferative capacity, poor cytotoxic molecules expression and, poor cytokine production ability). Interestingly, CD8^+^ T cells restricted by HLA-B*27/B*57 evade regulatory T cells (Tregs) suppression but not those restricted by non-HLA-B*27/B*57. CD8^+^ T cells restricted by non-HLA-B*27/B*57 when encountering their epitopes upregulate TIM-3 but not those restricted by HLA-B*27/B*57 and HLA-B*35Px. As a result, CD8^+^ T cells restricted by non-HLA-B*27/B*57 become suppressed by Tregs via TIM-3: Galectin-9 interactions. Strikingly, CD8^+^ T cells restricted by HLA-B*35Px upregulate CTLA-4 when encountering their epitopes, which render them to an exhausted phenotype. This differential response is linked to the up-regulation of Eomes, Blimp-1 but low T-bet expression in CD8^+^ T cells restricted by HLA-B*35Px. These results implicate that reinvigoration of these cells might be feasible using an anti-CTLA-4 antibody.

## Introduction

HIV-1 infection in the absence of antiretroviral therapy (ART) has taken millions of lives. This infection results in the progressive depletion of CD4^+^ T cells and progression to AIDS in the majority of patients without ART treatment[1]. However, the rate of disease progression is markedly different among subjects, with host genetic factors having a crucial impact on HIV-1 disease progression. For example, HLA-B*27 and B-*57 alleles are associated with slower progression to AIDS and are highly enriched in a rare group of HIV-infected individuals called Long-term nonprogressors (LTNPs) [2–4]. Conversely, HLA-B*35 and B*53 alleles are associated with the rapid disease progression to AIDS[5–7].

Antigen-specific cytotoxic T lymphocytes (CTLs) play a major role in viral control in HIV-1 infection, which explains the influence of HLA class I alleles on the rate of disease progression [8–10]. For example, the emergence of antigen-specific CTLs in HIV-infected individuals coincides with a reduction in the viral load [9], and the depletion of CD8^+^ T cells is associated with disease progression[11]. Several mechanisms have been described for the efficient control of HIV by HLA-B*27 and B-*57-restricted CD8^+^ T cells in LTNPs. For example, CTL restricted by protective HLA-B*27 and B-*57 alleles have superior proliferative capacity coupled with higher up-regulation of their cytotoxic granules compared to CTLs restricted by other HLA alleles [12,13]. In agreement, we have previously shown that CTLs restricted by HLA-B*27 and B-*57 have higher effector function capabilities, while the reverse was true for CTLs restricted by non-HLA-B*27 and B-*57[3]. We also described the mechanism associated with the differential functionality of CTLs depending on their HLA-allele restrictions. We found that CD8^+^ T cells restricted by HLA-B*27 and B-*57 evade regulatory T cells (Tregs)-mediated suppression while CD8^+^ T cells restricted by non-HLA-B*27/B*57 were suppressed by Tregs[3]. This was associated with the upregulation of the co-inhibitory receptor, TIM-3, which rendered them susceptible to Treg-medicated suppression via Galectin-9 (Gal-9): TIM-3 interactions[3]. Conversely, CTLs restricted by HLA-B*27/B*57 did not upregulate TIM-3 upon recognition of their cognate epitopes and therefore did not become suppressed by Tregs. Instead, they upregulated Granzyme B (GzmB) and continued to target virally infected cells and even Tregs[3]. However, the mechanism associated with the rapid progression to AIDS in individuals possessing HLA-B-*35/B*53 alleles has not been fully-studied. HLA-B*35 subtypes are classified to HLA-B*35-Px (e.g. HLA-B*35:02, HLA-B*35:03 and HLA-B*35:04) with a significant association with accelerated disease progression, whereas the other subtype defined as HLA-B*35Py (HLA-B*35:01 and HLA-B*35:08) lacks any association with the disease progression [14,15]. The effect of HLA-B-*35.01 allele on CTL functions has not been free of controversy but a recent study indicated that the Y135F mutation is the main factor for the detrimental effects of this HLA-allele on disease outcome(15), which can explain such differential effects in various cohorts [16]. The underlying mechanism of HLA-*B35Px and HIV disease progression in terms of immunological response is not well studied. Nevertheless, the binding of HLA-B*3503 to the inhibitory MHC I receptor immunoglobulin like-receptor 4 (ILT4) on dendritic cells (DCs) results in impaired DC function and suggested to be associated with the disease progression[17]. It appears that disease progression associated with HLA-B*35Px begins during the early stage of infection and remains noticeable[18]. This could be related to a deficient CTL response in HLA-B*35Px individuals compared to those with HLA-B*35Py[19]. Another explanation might be related to the inability of these alleles to present HIV epitopes to CTLs[20]. The HLA-B*53 allele is also included with HLA-B*35Px and disease progression because of its close phylogenic connection with HLA-B*35[6]. This implies that some features attributed to CTL’s response to HLA-B*35/B*53 restricted epitopes might contribute to the disease progression in patients having these HLA-alleles.

Another potential mechanism for the rapid disease progression in HLA-B*35Px could be explained by the dysfunctionality of CTLs restricted by these HLA-alleles. During chronic viral infections such as HIV-1 because of the chronicity of the hyperimmune activation, the program of CD8^+^ T cell differentiation is substantially altered[21]. This scenario is often associated with deteriorating CTL function, a state of dysfunction commonly called exhaustion[22]. Exhausted CD8^+^ T cells exhibit impaired effector functions such as cytokine production, proliferate capacity, and cytotoxicity. Instead, they manifest sustained upregulation and co-expression of multiple co-inhibitory receptors or immune checkpoints such as PD-1, CTLA-4, TIM-3, and other co-inhibitory receptors[22,23]. However, the dysfunctionality of antigen-specific CTLs restricted by HLA-B*35Px in terms of these immune checkpoints has never been well studied before. Therefore, in the present study, we first examined the functionality of CTLs restricted by HLA-B*35/B*53 in comparison with those restricted by HLA-B*27 and B-*57 and non- HLA-B*27 and B-*57 alleles. Then determined whether CTLs restricted by HLA-B*35Px are suppressed compared with those restricted by non- HLA-B*27 and B-*57 alleles and HLA-B-*27/B*57. Furthermore, we investigated the expression levels of PD-1, TIM-3, and CTLA-4 on antigen-specific CTLs following *in vitro* stimulation with their cognate epitopes. Finally, we measured mRNA levels for Eomes, Blimp-1, T-bet, IL-2, IFN-γ and TNF-α genes in antigen-specific CD8^+^ T cells restricted by HLA-B*35/*53 versus those CD8^+^ T cells restricted by HLA-B*27/B*57 alleles. Our cohort was consisting of only HLA-B35Px (HLA-B*35:02 and HLA-B*35:03 subtypes) and thus our focus is on HLA-B*35Px throughout this report. Our studies for the very first time, to our knowledge, provides a novel insight into the immune mechanisms associated with impaired control of HIV-1 in HLA-B*35Px patients.

## Results

### Diminished cytokine production by HLA-B*35-restricted CD8^+^ T cells

**Fig 1:**
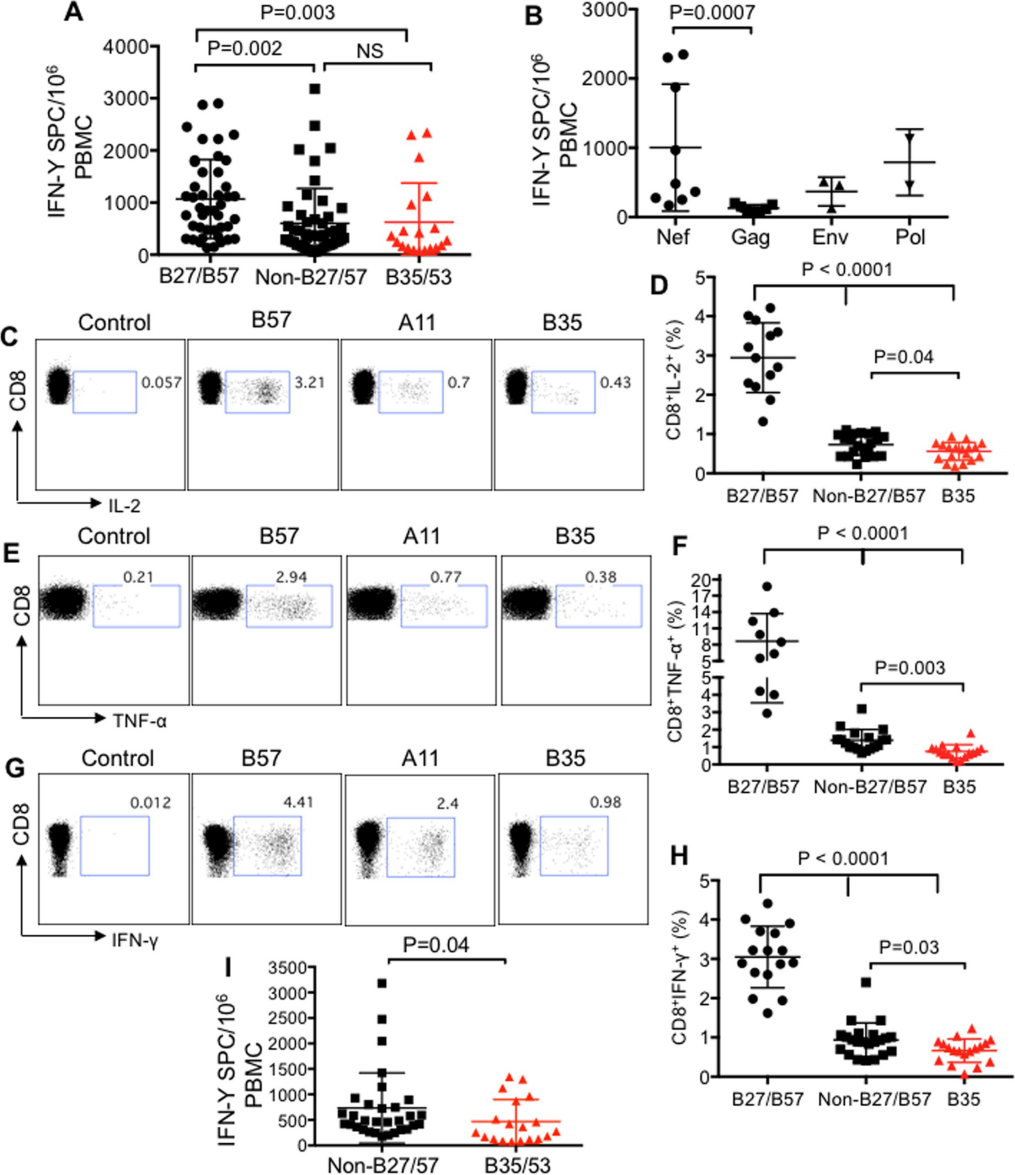
Diminished cytokine production by HLA-B*35Px-restricted CD8^+^ T cells. **(A)** Cumulative data showing quantification of IFN-γ secreting cells (spot count)/ 10^6^ PBMCs following stimulation with different 15 mer peptides restricted by HLA-B*27/B57, non-HLA-B*27/B57 (HLA-A*03, A*02, A*24, A*26 and B*40) and HLA-B*35Px/B*53 using ELISpot assay. (**B**) Cumulative data showing the breadth of IFN-γ response to different HIV-proteins in PBMCs of HLA-B*35Px individuals using ELISpot assay. (**C)** Representative flow cytometry plots, and (**D**) cumulative data showing percentages of IL-2 secreting CD8^+^ T cells restricted by HLA-B*27/B57, non-HLA-B*27/B57 and HLA-B*35 in HIV-infected individuals following stimulation with their cognate epitopes (2 μg/ml) for 72 hrs as measured by ICS. (**E)** Representative flow cytometry plots, and (**F**) cumulative data showing percentages of TNF-α secreting CD8^+^ T cells restricted by HLA-B*27/B57, non-HLA-B*27/B57 and HLA-B*35 in HIV-infected individuals following stimulation of PBMCs with their cognate epitopes (2 μg/ml) for 72 hrs using ICS. (**G**) Representative flow cytometry plots, and (**H**) cumulative data showing percentages of IFN-γ secreting CD8^+^ T cells restricted by HLA-B*27/B57, non-HLA-B*27/B57 and HLA-B*35 in HIV-infected individuals following stimulation with their cognate epitopes (2 μg/ml) for 72 hrs as measured by ICS. (**I)** Cumulative data showing quantification of IFN-γ secreting cells (spot count)/ 10^6^ PBMCs following stimulation with different epitopes restricted by non-HLA-B*27/B57 (HLA-A*03, A*01, A*02, A*24, A*11, B*38, B*08 and B*44) and HLA-B*35Px/B*53 from 5 HLA-B35/B53 individuals using ELISpot assay. Each point represents data from an epitope. Bar, mean ± one standard error.

For these studies, we used PBMCs from 10 LTNPs defined as individuals who have been infected with HIV more than 11 years, ART-naïve, with CD4 count > 400 and viral load <1000 copies/ml (S1 Table). In addition, we identified 5 HIV-1 infected individuals with HLA-B*35Px who have been infected with HIV > 1 year, ART-naïve, 400 > CD4 count with high viral load (> 65,000 copies/ml) (S1 Table). The initial epitope mapping using ELISpot assay indicated a greater breadth of CTL response in HLA-B*35Px restricted individuals (5–13 epitopes, an average of 9.4) compared with those with HLA-B*27/B*57 (3–8 epitopes, an average of 5.4) S2 and S3 Tables respectively. To assess the functionality of HIV-specific CD8^+^ T cells restricted by HLA-B*35 versus HLA-B*27/B*57 and non-B*27/B*57 (e.g. HLA-A*03, A*02, A*24, A*26, B*40), we measured their IFN-γ secretion ability following *in vitro* stimulation of PBMCs with their recognizing 15 mer peptides using ELISpot. In response to peptide stimulation, antigen-specific CD8^+^ T cells restricted by HLA-B*35Px/B*53 produced significantly lesser IFN-γ compared to CD8^+^ T cells restricted by HLA-B*27/B*57 but not compared to non- HLA-B*27/B*57 alleles (Fig 1A). Besides, we observed a significantly lower IFN-γ secretion by CD8^+^ T cells restricted by non-HLA-B*27/B*57 than HLA-B*27/B*57 alleles (Fig 1A). We found CD8^+^ T cells restricted by HLA-B*35Px target different parts of the virus including *Nef*, Gag, *Env*, and *Pol*. However, the *Nef* protein elicited the most dominant CD8^+^ T cell response [24], as illustrated by significantly higher IFN-γ response compared to CTLs recognizing the Gag protein (Fig 1B).

To further characterize the functionality of HIV epitope-specific CD8^+^ T cells, we decided to examine their functionality in terms of IL-2, IFN-γ, and TNF-α production following *in vitro* stimulation with different cognate epitopes using intercellular cytokine staining (ICS). PBMCs were stimulated for ~ 66 hours, before the addition of the Golgi blocker for an additional 6 hrs. We observed significantly higher production of IL-2, IFN-γ, and TNF-α by CD8^+^ T cells restricted by HLA-B*27/B*57 compared with CD8^+^ T cells restricted by non-B*27/B*57 and HLA-B*35Px (Fig 1C–1H). Interestingly, we found that CD8^+^ T cells restricted by HLA-B*35Px produced significantly lesser IL-2, IFN-γ, and TNF-α than CD8^+^ T cells restricted by non-B*27/B*57 HLA-alleles (Fig 1C–1H). Specifically, CD8^+^ T cells restricted by HLA-B*35/B*53 exhibited impaired cytokine production capabilities when compared with their counterparts restricted by non-HLA-B*27/B*57 within the same individuals (S1A–S1C Fig). Such dysfunctionality may explain the association of HLA-B*35Px alleles (HLA-B*3502, HLA-B*3503, and HLA-B*3504) but not HLA-B*35-Py alleles (HLA-B*3501 and HLA-B*3508) with rapid disease progression [14,25]. However, the discrepancy in the ELISpot assay (Fig 1A) with the ICS assay might be related to using 15 mer peptides in ELISpot versus 8–11 mer epitopes in the ICS assay. Using 8–11 mer epitopes in ELISpot assay confirmed that CD8^+^ T cells restricted by HLA-B*35Px exhibit impaired IFN-γ secretion capabilities compared to those restricted by non-HLA-B*27/B*57 within individuals restricted by HLA-B*35Px (Fig 1I).

### HLA-B*35Px/B*53-restricted CD8^+^ T cells have impaired expression of perforin, GzmB and lower proliferative capacity

**Fig 2:**
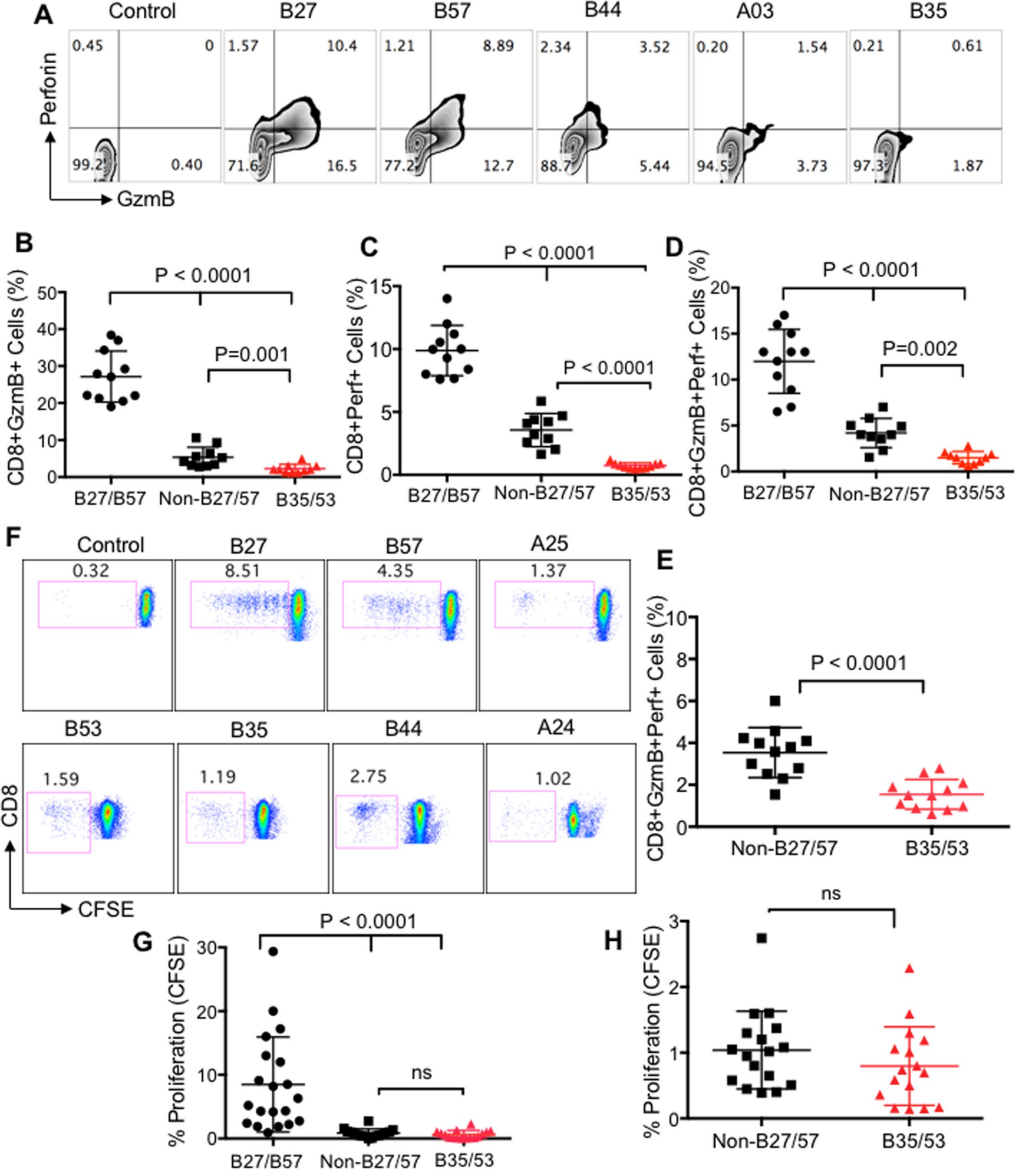
HLA-B*35/53-restricted CD8^+^ T cells have impaired expression of perforin, GzmB with low proliferative capacity. **(A-D**) Representative plots and cumulative data showing expression of GzmB, perforin, and their co-expression respectively, in CD8^+^ T cells restricted by HLA-B*27/B57, non-HLA-B*27/B57 and HLA-B*35Px/B*53 in HIV-infected individuals following stimulation of PBMCs with their cognate epitopes (2 μg/ml) for 72 hrs using ICS. (**E**) Cumulative data showing co-expression of GzmB/perforin in CD8^+^ T cells restricted by HLA-B*27/B*57 versus those restricted by HLA-B*35/B*53 within individuals having HLA-B35Px. (**F**) Representative plots, and (**G**) cumulative data indicating percentages of proliferated CD8^+^ T cells restricted by different HLA-alleles following stimulation with their cognate epitopes (2 μg/ml) for 96 hrs measured by CFSE titration assay. (**H**) Cumulative data showing percentages of proliferated CD8^+^ T cells restricted by HLA-B*27/B*57 versus those restricted by HLA-B*35/B*53 within individuals having HLA-B35Px. Each point represents data from an epitope. Bar, mean ± one standard error.

It has been shown that the quality of CD8^+^ T cell response as measured by the production of different cytokines and cytotoxic molecules better correlates with HIV disease control [13]. In particular, the cytotoxic granule-mediated cell death by CTLs requires the synergic action of both perforin and GzmB[26]. Thus, we examined the expression of perforin, GzmB and co-expression of these cytotoxic molecules in CTLs restricted by different HLA-alleles upon stimulation with their cognate epitopes. Similar to the cytokine production, we observed significantly lower expression of GzmB, perforin and their co-expression in CD8^+^ T cells restricted by HLA-B*35Px/B*53 compared to their counterparts restricted by either non-HLA-B*27/B57 or HLA-B*27/B*57 (Fig 2A–2D). Consistent with what we have previously shown[3], we found that CD8^+^ T cells restricted by HLA-B*27/B*57 expressed significantly higher GzmB, perforin, and GzmB/perforin than CD8^+^ T cells restricted by non-HLA-B*27/B*57 (Fig 2A–2D). In contrast, CD8^+^ T cells restricted by HLA-B*35/B*53 expressed significantly lower levels of GzmB/perforin compared to those restricted by HLA-B*27/B*57 and non-HLA-B*27/B*57 (Fig 2A and 2D). Within those individuals with HLA-B*35Px, CD8^+^ T cells restricted by HLA-B*35/B*53 showed impaired GzmB/perforin expression compared to their siblings restricted with non-HLA-B*27/B*57 (Fig 2E). These observations indicate that CD8^+^ T cells restricted by HLA-B*35Px/B*53 exhibit dysfunctional effector functions when recognizing their cognate epitopes *in vitro*.

Also, we measured the proliferative capacity of CD8^+^ T cells restricted by different HLA-alleles using CFSE assay according to our previous reports[23,27]. We observed that epitope-specific CD8^+^ T cells restricted by HLA-B*35Px/B*53 had substantially lower proliferation capacity compared to CD8^+^ T cells restricted by HLA-B*27/B*57 alleles (Fig 2F and 2G). However, we didn’t observe any significant difference between the proliferative capacity of HLA-B*35Px/B*53-restricted CD8^+^ T cells compared to those restricted with non-HLA-B*27/B*57 alleles within all patient groups (Fig 2F and 2G) or when specifically assessed in those individuals having HLA-B*35/B*53 (Fig 2H). These data indicate that although CD8^+^ T cells restricted by non-HLA-B*27/B*57 have higher cytolytic molecules contents than HLA-B*35Px/B*53, both groups exhibited significant impairment proliferation capacity compared to CD8^+^ T cells restricted by HLA-B*27/B*57.

### The impaired proliferative capacity of HLA-B*35Px-restricted CD8^+^ T cells is not related to Tregs-mediated suppression

**Fig 3:**
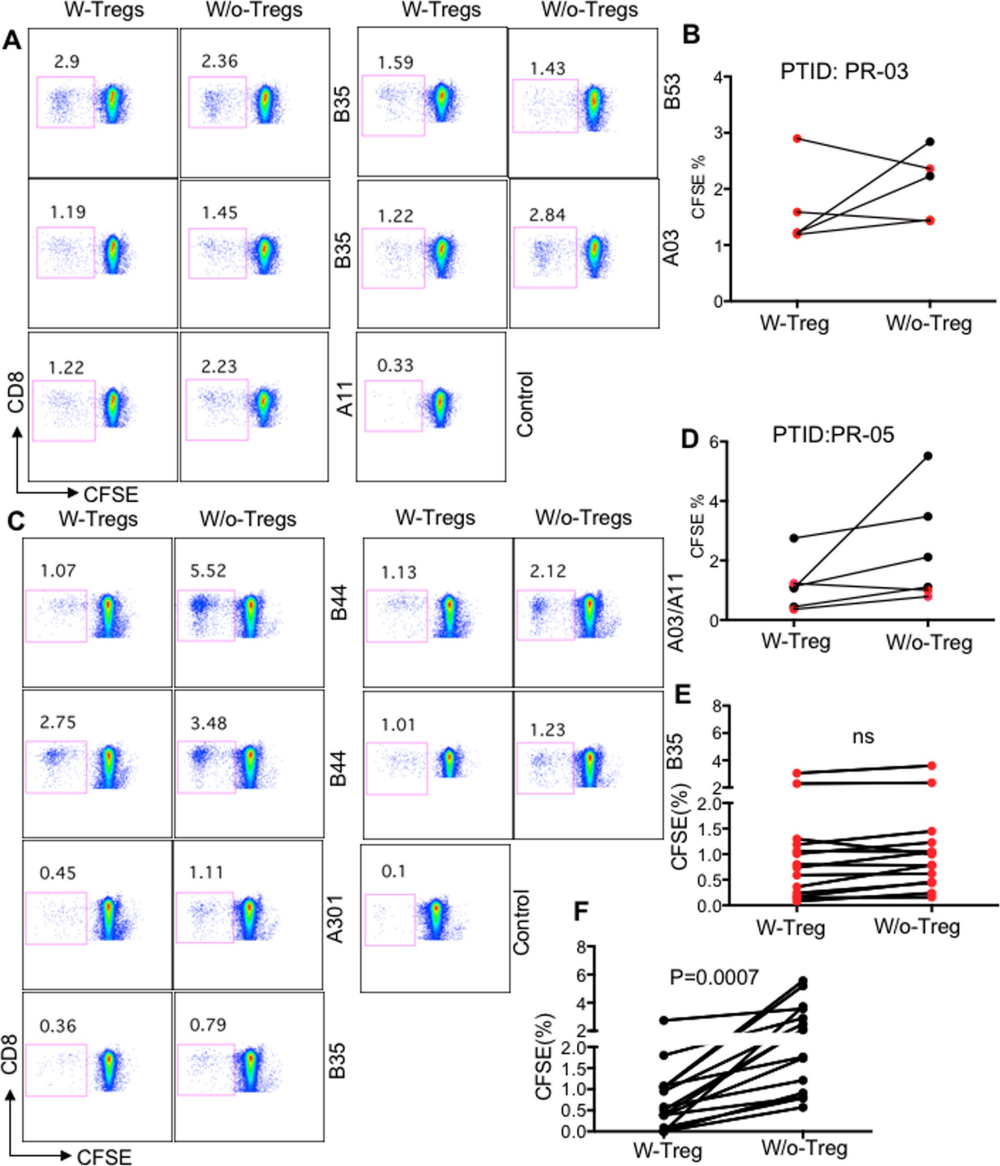
The proliferation of HLA-B*35-restricted CD8^+^ T cells is not suppressed by Tregs. **(A-B**) Representative plots showing proliferation of CD8^+^ T cells restricted by different HLA-alleles within an HLA-B*35Px patient (PTID:PR-03) following stimulation with their cognate epitopes in the presence or absence of Tregs for 96 hrs. Red symbols representing CD8^+^ T cells restricted by HLA-B*35/B*53 and black symbols those restricted by HLA-A*03/A*11. (**C-D**) Representative plots showing proliferation of CD8^+^ T cells restricted by different HLA-alleles within an HLA-B*35Px patient (PTID:PR-05) following stimulation with their cognate epitopes (2 μg/ml for 96 hrs) in the presence or absence of Tregs. (**E**) Cumulative data showing percentages of proliferated antigen-specific CD8^+^ T cells restricted by HLA-B*35Px following stimulation with their cognate epitopes. Red symbols representing CD8^+^ T cells restricted by HLA-B*35 and black symbols those restricted by HLA-A*03/A*11, HLA-B*44 and HLA-A*301. (**F**) Cumulative data showing percentages of proliferated antigen-specific CD8^+^ T cells restricted by HLA-A*01, A*02, A*03, A*08, A*11, A*24, B*38 and B*44 following stimulation with their cognate epitopes. Each point represents data from an epitope.

Previously, we have shown differential susceptibility of CTLs to Tregs-medicated suppression within a single individual depending on their HLA-allele restriction. We found the proliferation of CTLs restricted by non-HLA-B*27/57 alleles was suppressed by Tregs, whereas CTLs restricted by HLA-B*27/B57 alleles were resistant to Tregs-mediated suppression [3,4]. Although the magnitude of CTL response in non-HLA-B*27/B57 appeared to be lower than their HLA-B*27/B57 counterparts, we have shown that the differential suppression was independent of CTL frequency[3]. To determine whether impaired effector functions in CTLs restricted by HLA-B*35Px was associated with Tregs-mediated suppression, we investigated the proliferative capacity of CD8^+^ T cells restricted by HLA-B*35Px following stimulation with their cognate epitopes using proliferation assay in the presence and absence of autologous Tregs. Interestingly, we observed that the proliferation of CD8^+^ T cells restricted by HLA-B*35Px/B53 alleles was not suppressed by Tregs (Fig 3A–3D). We extended our analysis to compare the proliferative capacity of CD8^+^ T cells restricted by non- HLA-B*27/B57 versus HLA-B*35/B53-restricted alleles within each individual. Inconsistent with our previous report [3], we observed an enhanced proliferative capacity of CD8^+^ T cells restricted by non-HLA-B*27/B57 in the absence of Tregs (Fig 3A–3D). In contrast, the removal of Tregs from PBMCs did not enhance the proliferative capacity of CD8^+^ T cells restricted by HLA-B*35Px/B53 (Fig 3A–3D). These observations suggest that CD8^+^ T cells restricted by HLA-B*35Px/B53 have impaired proliferative capabilities regardless of Tregs presence (Fig 3E), which is in sharp contrast with CD8^+^ T cells restricted by non-HLA-B*27/B57 (Fig 3F). Therefore, differential functionality of CD8^+^ T cells restricted by different HLA-alleles in the absence of Tregs within the same individual suggests the presence of other potential extrinsic or intrinsic mechanisms.

### Cytokine production ability of CD8^+^ T cells restricted by HLA-B*27/B57 and HLA-B*35Px is not influenced by Tregs whereas the opposite is true for non-HLA-B*27/B57 restricted CTLs

**Fig 4:**
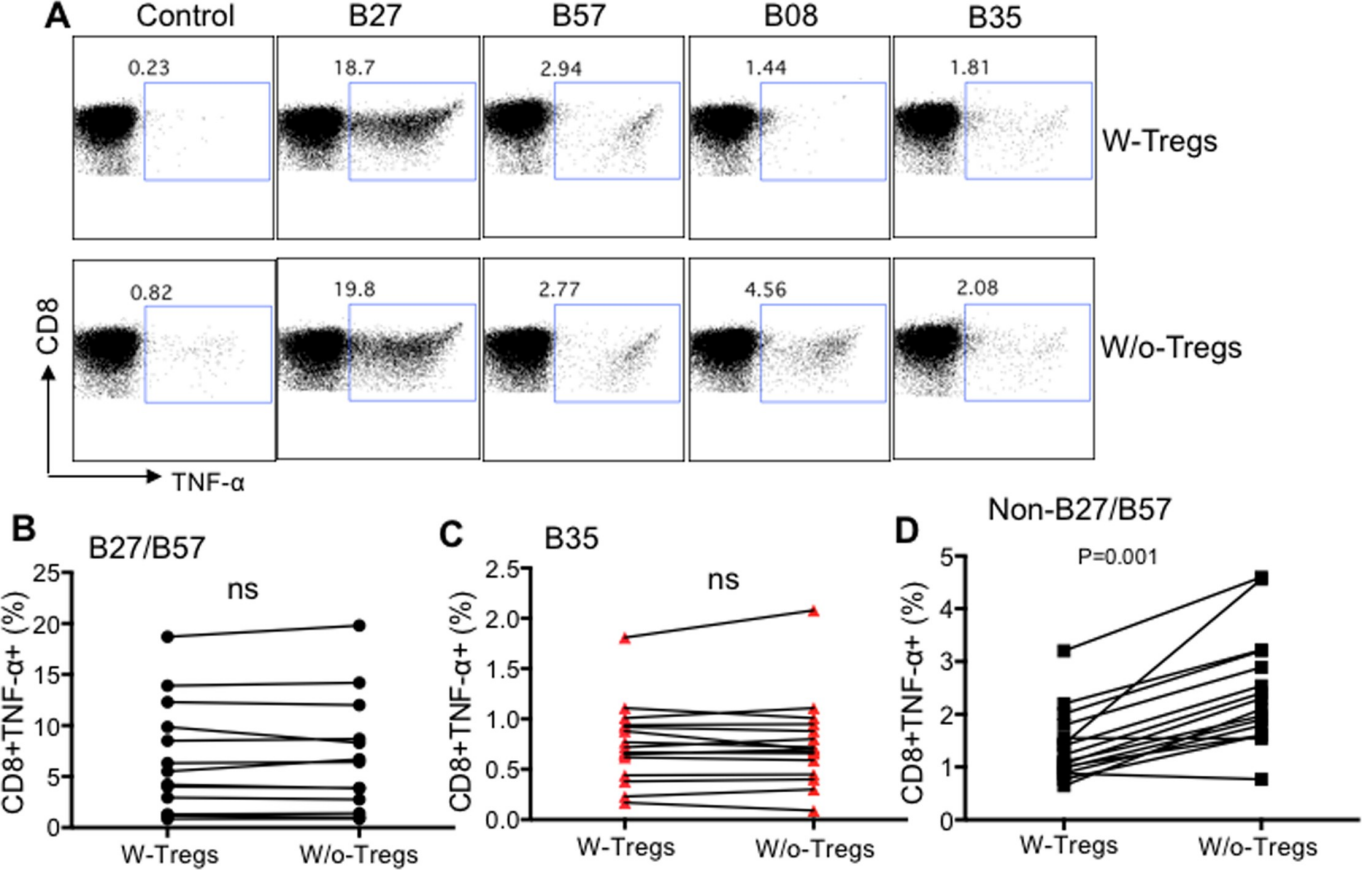
Differential suppression of TNF-α by Tregs depending on the HLA-allele restriction of CTLs. **A**) Representative flow cytometry plots of % TNF-α production by CD8^+^ T cells restricted by different HLA-alleles, as shown, following stimulation with their cognate epitopes (2 μg/ml for 72 hrs) measured by ICS in the presence or absence of autologous Tregs. **B-D**) Cumulative data showing %TNF-α secreting CD8^+^ T cells restricted by either HLA-B*27/B57, HLA-B*35Px or non-HLA-B*27/57 following stimulation with their cognate epitopes (2 μg/ml) for 72 hrs as measured by ICS. Each point represents data from an epitope.

In addition to the proliferative capacity of CTLs restricted by different HLA-alleles, we decided to determine whether Tregs modulate cytokine production capabilities of CD8^+^ T cells restricted by different HLA-alleles. We found similar to proliferation, cytokine production ability (e.g. TNF-α) of CD8^+^ T cells restricted by HLA-B*27/B57 and HLA-B*35Px remained unchanged in the presence/absence of Tregs when PBMCs were stimulated with their cognate epitopes for 72 hrs (Fig 4A–4C). In contrast, recognition of cognate epitopes by CD8^+^ T cells restricted by non-HLA-B*27/B57 in the absence of Tregs unleashed TNF-α production (Fig 4A–4D). These results indicate that CD8^+^ T cells restricted by HLA-B*27/B57 evade Tregs mediated suppression but those restricted by HLA-B*35 exhibit impaired functionality regardless of Tregs presence.

### Impaired proliferative capacity of HLA-B*35Px*-*restricted CD8^+^ T cells is not mediated through Tim-3 and PDL-1 but CTLA-4

**Fig 5:**
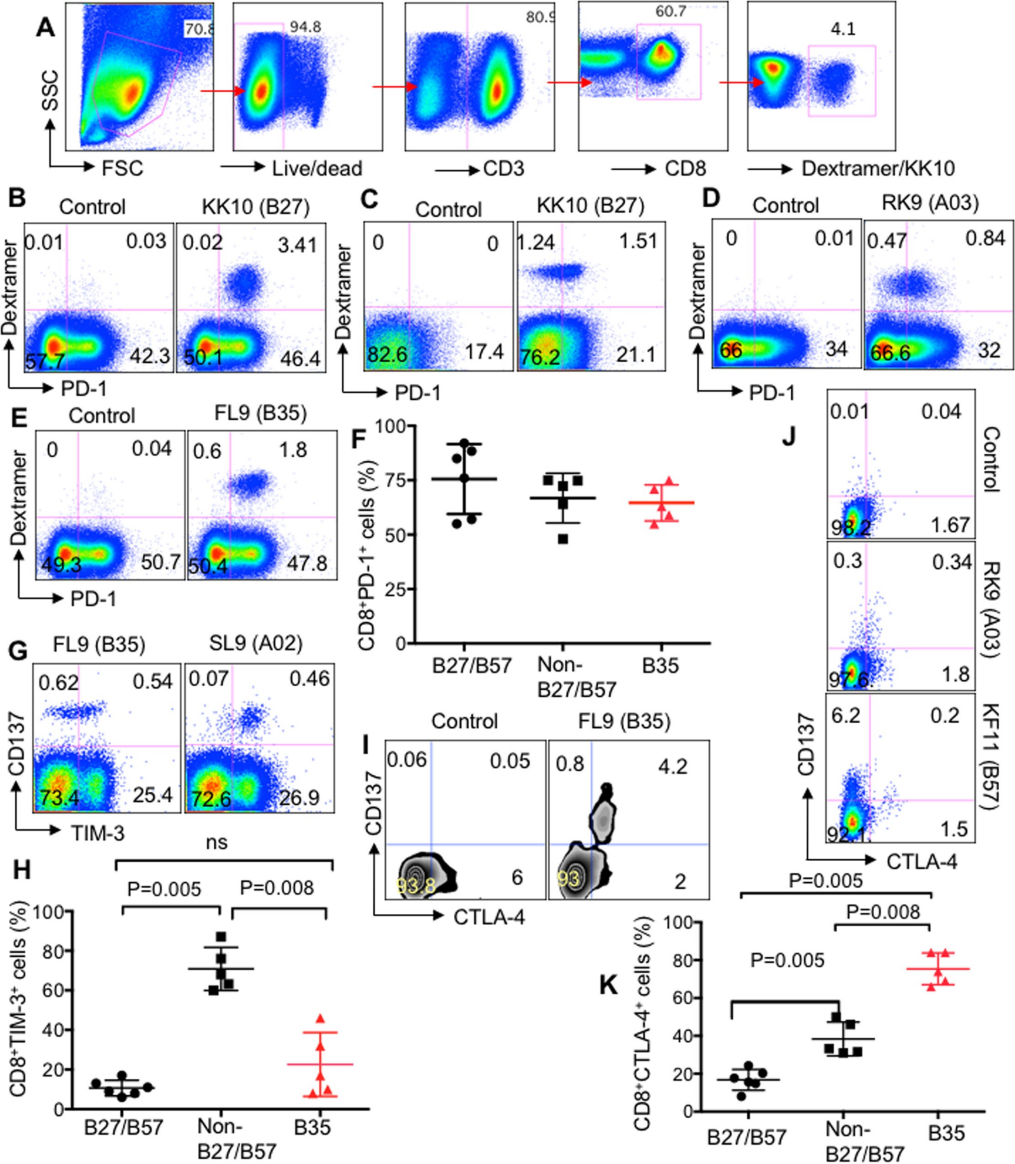
Differential expression of co-inhibitory receptors by CD8^+^ T cells upon recognition of their cognate epitopes *in vitro*. (**A**) Representative plots showing gating strategy for identification of antigen-specific T cells using tetramer staining. (**B**, **C**) Representative flow cytometry plot showing % co-expression of Tetramer^+^PD-1^+^ CD8^+^ T cells following stimulation of PBMCs with B27-KK10 (KRWIILGLNK) epitope (2 μg/ml) for 72 hrs in patient #LNP01 and patients #LNP03 respectively. **(D, E**) Representative flow cytometry plot showing % co-expression of Tetramer^+^PD-1^+^ CD8^+^ T cells following stimulation of PBMCs with A03-RK9 (RLRPGGKKK) and B35-FL9 (FPVKPQVPL) epitopes (2 μg/ml) for 72 hrs in patient #PR03 and patients #PR01 respectively. (**F**) Cumulative data showing % tetramer^+^PD-1^+^ in CD8^+^ T cells restricted by different HLA-alleles, as shown, following encounter with their cognate epitopes for 72 hrs. **(G)** Representative flow cytometry plot showing % co-expression of Tetramer^+^TIM-3^+^ CD8^+^ T cells following stimulation of PBMCs with A02-SL9 (SLYNTVATL) and B35-FL9 (FPVKPQVPL) epitopes (2 μg/ml) for 72 hrs in patients #PR04 and #PR01, respectively. (**H**) Cumulative data showing % TIM-3 expressing antigen-specific CD8^+^ T cells following stimulation with their cognate epitopes for 72 hrs. (**I**, **J**) Representative flow cytometry plot showing % co-expression of Tetramer^+^CTLA-4^+^ CD8^+^ T cells following stimulation of PBMCs with B35-FL9 (FPVKPQVPL), A03-RK9 (RLRPGGKKK), and B57-KF-11 (KAFSPEVIPMF) epitopes (2 μg/ml) for 72 hrs in patients #PR01 and #PR03 and #LNP02, respectively. (**K**) Cumulative data showing % TIM-3 expressing antigen-specific CD8^+^ T cells following stimulation with their cognate epitopes for 72 hrs. Each point represents data from an epitope. Bar, mean ± one standard error.

Upon exposure to their cognate epitopes, CD8^+^ T cells upregulate the expression of co-inhibitory receptors, such as PD-1, TIM-3, and CTLA-4 to self-limit their activation [3,22,23]. However, chronic upregulation of co-inhibitory receptors and engagement with their corresponding ligands on antigen-presenting cells (APCs) or Tregs results in the impairment of CTLs functions [22,28]. Previously, we showed that CD8^+^ T cells restricted by non-HLA-B*27/B57 upon stimulation with their cognate epitopes upregulate TIM-3 and subsequently become suppressed by Gal-9 expressing Tregs[3]. In contrast, CD8^+^ T cells restricted by HLA-B*27/B57 within the same individual do not upregulate TIM-3 upon stimulation with their cognate epitopes and therefore evade Tregs-mediated suppression via TIM-3: Gal-9[3]. To determine the expression of co-inhibitory receptors on antigen-specific CTLs, we stimulated PBMCs with various epitopes restricted by HLA-B*35Px, non-HLA-B*27/B57 and HLA-B*27/B57 for 72 hrs and then measured the frequency of PD-1, TIM-3, and CTLA-4 expressing CTLs compared to unstimulated wells. We acquired >600,000 cells/sample for the identification of antigen-specific CTLs either using tetramers (Fig 5A) or CD137. Due to the unavailability of tetramers, in some experiments, we used antigen-triggered CD137 to identify antigen-specific CD8^+^ T cells as we have reported elsewhere[3]. We found that CD8^+^ T cells restricted by HLA-B*27/B57 upregulate substantial levels of PD-1 but it appeared to be donor-dependent, the different expression level of PD-1 was observed for the same epitope (e.g. KK10) in different patients (Fig 5B and 5C). It appears that CD8^+^ T cells restricted by either non-HLA-B*27/B57 or HLA-B*35Px also upregulate PD-1 when encountering their cognate epitopes (Fig 5D and 5E), respectively. Overall, antigen-specific CD8^+^ T cells regardless of their HLA-allele restriction upregulate substantial levels of PD-1 (Fig 5F). Although we have shown elevated levels of TIM-3 on CTLs restricted by non-HLA-B*27/B*57 compared with HLA-B*27/B*57[3], surprisingly, the expression of this co-inhibitory receptor was significantly lower on CD8^+^ T cells restricted by HLA-B*35Px compared to those restricted by non-HLA-B*27/B*57 upon stimulation with their cognate epitopes for 72 hrs (Fig 5G and 5H). However, there was no significant difference between the frequency of TIM-3+CD8^+^ T cells restricted by HLA-B*27/B*57 versus HLA-B*35Px (Fig 5H). Besides, we measured the frequency of CTLA-4 expressing antigen-specific CD8^+^ T cells following stimulation of PBMCs with different cognate epitopes restricted by different HLA-alleles. We found that CD8^+^ T cells restricted by HLA-B*35Px expressed substantially higher levels of CTLA-4 compared to the other HLA-groups (Fig 5I–5K).

**Fig 6:**
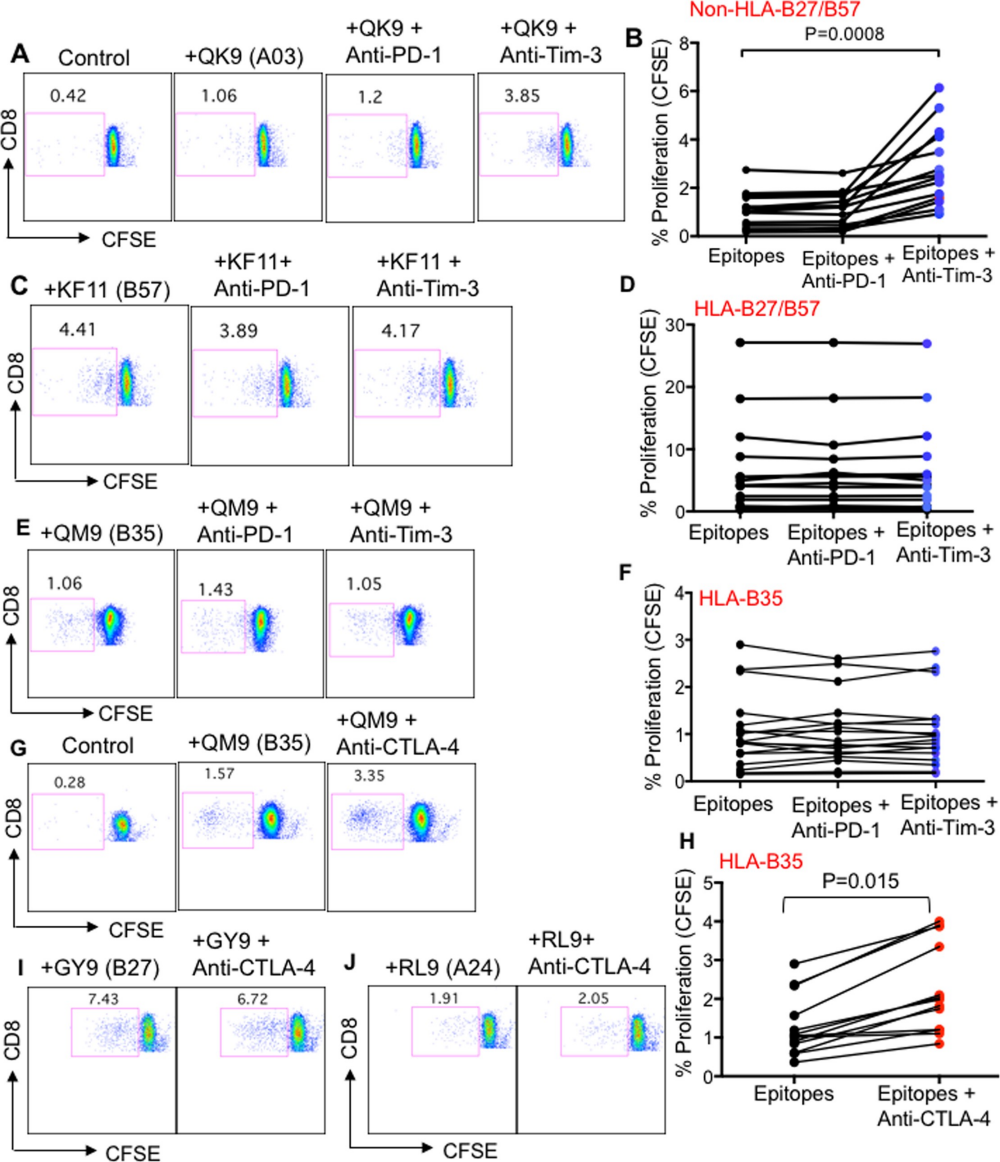
Differential effects of anti-TIM-3, anti-PD-1 and anti-CTLA-4 blocking antibodies on rescuing of the proliferative capacity of CD8^+^ T cells restricted by different HLA-alleles. **(A**) Representative flow cytometry plots, and (**B**) cumulative data showing proliferation of antigen-specific CD8^+^ T cells restricted by non-HLA-B*27/B57 following stimulation with their cognate-epitopes in the absence or presence of anti-PD-1 (Pembrolizumab 5 μg/ml), anti-TIM-3 (10 μg/ml LEAF) antibodies for 96 hours as measured by CFSE dilution assay, QK9 (QVPLRPMTYK), patient #LNP04. (**C**) Representative flow cytometry plots, and (**D**) cumulative data showing proliferation of antigen-specific CD8^+^ T cells restricted by HLA-B*27/B57 following stimulation with their cognate-epitopes in the absence or presence of anti-PD-1 (Pembrolizumab 5 μg/ml), anti-TIM-3 (10 μg/ml LEAF) antibodies for 96 hours as measured by CFSE dilution assay, KF11 (KAFSPEVIPMF), patient #LNP04. (**E**) Representative flow cytometry plots, and (**F**) cumulative data showing proliferation of antigen-specific CD8^+^ T cells restricted by HLA-B*35Px following stimulation with their cognate-epitopes in the absence or presence of anti-PD-1 (Pembrolizumab 5 μg/ml), anti-TIM-3 (10 μg/ml LEAF) antibodies for 96 hours as measured by CFSE dilution assay, QM9 (QVTNSATIM), patient #PR05. (**G**) Representative flow cytometry plots, and (**H**) cumulative data showing proliferation of antigen-specific CD8^+^ T cells restricted by HLA-B*35Px following stimulation with their cognate-epitopes in the absence or presence of anti-CTLA-4 (Ipilimumab 5 μg/ml) antibody for 96 hours as measured by CFSE dilution assay, QM9 (QVTNSATIM), patient #PR05. (**I**) Representative flow cytometry plots showing proliferation of antigen-specific CD8^+^ T cells restricted by HLA-B*27 following stimulation with GY9 (GLNKIVRMY) epitope, and (**J**) CD8^+^ T cells restricted by HLA-A24 following stimulation with epitope RL9 (RYLKDQQLL) in the absence or presence of anti-CTLA-4 (Ipilimumab 5 μg/ml) antibody for 96 hours as measured by CFSE dilution assay, patient #LNP01. Each point represents data from an epitope.

To investigate whether impaired functionality of HLA-B*35Px*-*restricted CD8^+^ T cells was mediated through the interaction of these co-inhibitory receptors with their corresponding ligands on APCs, we stimulated CFSE labeled PBMCs from different patients with various cognate epitopes in the presence or absence of blocking antibodies against PD-1, Pembrolizumab (5 μg/ml), CTLA-4 Ipilimumab (5 μg/ml) and TIM-3 (clone F38-2E2) according to our previous reports [29,30] for 96 hrs. Interestingly, we found that blocking PD-1: PDL-1 interactions had no substantial impact on CD8^+^ T cell proliferation regardless of their HLA-allele restriction (Fig 6A–6F). We observed the anti-TIM-3 antibody enhanced the proliferation of antigen-specific CD8^+^ T cells restricted by non-HLA-B*27/B57 but it did not enhance the proliferative capacity of CD8^+^ T cells restricted by HLA-B*27/B57 and HLA-B*35Px (Fig 6A–6F). Furthermore, we found that the anti-CTLA-4 blocking antibody significantly improved the proliferative capacity of CD8^+^ T cells restricted by HLA-B*35Px (Fig 6G and 6H) but not others (Fig 6I and 6J). Our results suggest that higher CTLA-4 expression on CD8^+^ T cells restricted by HLA-B*35Px might be an explanation for their impaired functionality.

### HLA-B*35Px restricted CD8^+^ T cells express high Eomes and Blimp-1 but low T-bet

**Fig 7:**
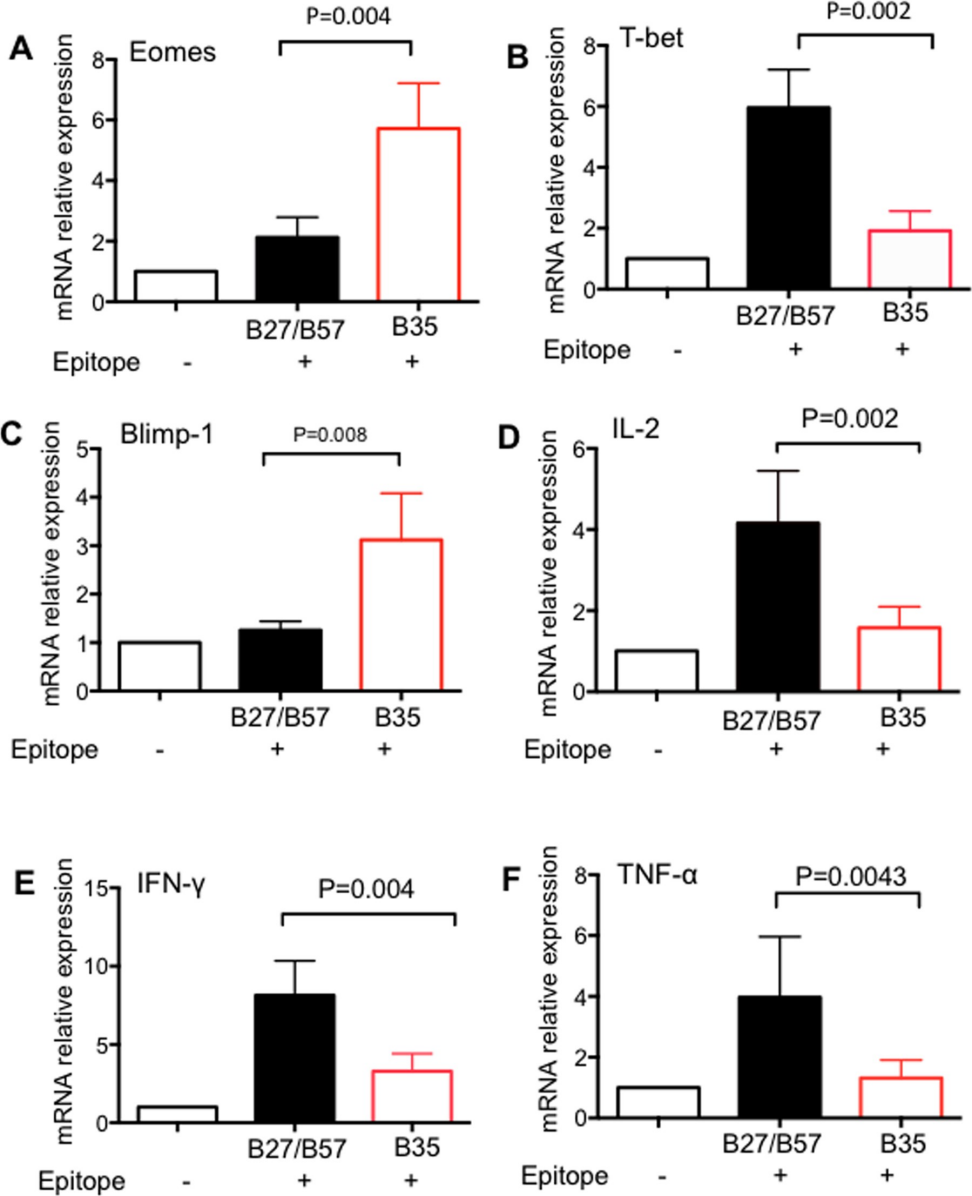
HLA-B*35Px restricted CTLs exhibit an exhausted phenotype at the gene level. **(A**) Relative mRNA expression of Eomes, (**B)** T-bet, (**C)** blimp-1, (**D)** IL-2, **E)** IFN-γ and **F**) TNF-α in CD8^+^ T cells restricted by HLA-B*35Px versus those restricted by HLA-B*27/B57 as quantified by qPCR. Data are from a minimum of five antigen-specific CD8^+^ T cells restricted by each HLA-type and isolated by sorting for RNA isolation and qPCR. Bar, mean ± one standard error.

T cell exhaustion is followed by transcriptional programming characterized by changes in the expression of several genes. For example, it has been shown that high expression of Eomes and low expression of T-bet are accompanied by up-regulation of co-inhibitory receptors and impaired functionality of HIV-specific CD8^+^ T cells [31]. As such, we performed qPCR to determine the expression levels of different genes in sorted antigen-specific CD8^+^ T cells restricted by HLA-B*35Px and those restricted by HLA-B*27/B57. We found that HLA-B*35*-*restricted CD8^+^ T cells expressed higher expression of Eomes but lower expression of T-bet mRNA compared to their counterparts restricted by HLA-B*27/B57-restricted CD8^+^ T cells (Fig 7A and 7B). We also observed a higher expression of Blimp-1, another gene associated with T cell exhaustion [32], in CD8^+^ T cells restricted by HLA-B*35Px comparted with HLA-B*27/B57-restricted CD8^+^ T (Fig 7C). To confirm our ICS results showing lower production of IL-2, IFN-γ, and TNF-α by HLA-B*35Px restricted CD8^+^ T cells once stimulated with their cognate epitopes. We examined the expression of mRNA for the above cytokines in HLA-B*35Px restricted CD8^+^ T cells versus those restricted by HLA-B*27/B57 antigen stimulation with their cognate epitopes overnight. Our results indicated decreased expression of mRNA for IL-2, IFN-γ, and TNF-α cytokines in CD8^+^ T cells restricted by HLA-B*35Px comparted with HLA-B*27/B57 restricted CD8^+^ T cells, hence confirming their cytokine production impairment at the gene level (Fig 7D–7F).

## Discussion

The association of HLA-*B35Px with HIV rapid disease progression has been well documented[5,7, 14,20]. Several studies have attributed impaired viral clearance to high mutation of epitopes recognized by HLA-*B35Px- restricted CTLs[14]. However, the mechanism(s) underlying the detrimental effect of this HLA-allele on disease outcome in HIV-1 infected individuals is not well understood. To investigate the potential mechanism(s), we first sought to identify HLA-B*35Px restricted HIV-1-specific epitopes recognized by CD8^+^ T cells in PBMCs of different patients in our cohort using IFN-γ ELISpot assay. First, we noticed that *Nef* protein elicited the most dominant CD8^+^ T cell response in these patients, which is consistent with other reports[24,33]. It has been shown that there is no difference in terms of IFN-γ production between CD8^+^ T cells restricted by HLA-B*35Px and Py_,_ however, the magnitude of IFN-γ response to these epitopes was shown to be very low (≤ 200 CFUs/10^6^ cells)[34]. In contrast, we observed a higher quantity of IFN-γ secreting CD8^+^ T cells upon recognition of HLA-B*35Px restricted epitopes in our cohort. To better understand the effector functions of CD8^+^ T cells restricted by HLA-B*35Px, we investigated multiples aspects of their functionality in comparison with CD8^+^ T cells restricted by protective (HLA-B*27/B*57*)* and non-protective HLA-alleles (e.g. HLA-A*01, A*02, A*03, A*11, A*24, A*26, B*40, B*08, B*38, B*44). Consistent with previous studies[12], we found that epitope-specific CD8^+^ CTL restricted by the protective HLA alleles (HLA-B*27/B*57) had significantly higher IFN-γ response compared to those restricted by non-protective alleles and HLA-B*35Px. The magnitude of IFN-γ production was significantly lower in PBMCs of patients restricted by HLA-B*35Px compared to those restricted by non-HLA-B*27/B*57 when stimulated with their cognate epitopes and examined by ELISpot. Similarly, HLA-B*35Px*-*restricted CD8^+^ T cells exhibited an impaired phenotype in terms of IL-2, TNF-α and IFN-γ production compared to non-HLA-B*27/B*57-restricted CD8^+^ T cells when encountered their cognate epitopes *in vitro*, as measured by ICS. However, there was no significant difference in the magnitude of IFN-γ producing cells between CD8^+^ T cells restricted by non-HLA-B*27/B*57 when stimulated with 15 mer peptides. This discrepancy might be related to using different stimulation methods e.g. 15 mer peptides versus 8–11 mer epitopes. In contrast to the epitope, the 15 mer peptide requires to be processed and presented by antigen-presenting cells (APCs), which may differentially influence CTL response. These findings are consistent with other studies showing polyfunctionality of CTLs restricted by HLA-B*27/B*57 alleles[3,12,13,35]. However, this is not in line with a report showing the lack of difference in cytokine production and degranulation between HLA-B*57 and HLA-B*35 carriers[33]. One possible explanation for the discrepancy in cytokine response would be that CTLs respond differently to individual epitopes that the peptide pools used in the previous study. Furthermore, we found that CD8^+^ T cells restricted by HLA-B*35Px had impaired expression of cytotoxic molecules GzmB and perforin. The cytotoxic granule-mediated cell apoptotic by CD8^+^ T cells requires the synergic action of both perforin and GzmB [26]. Nevertheless, CD8^+^ T cells restricted by HLA-B*35Px exhibited an impairment phenotype in terms of co-expression of these cytotoxic molecules. In contrast, the superiority of CD8^+^ T cells restricted by protective HLA-B*27/B57 alleles in terms of cytolytic molecules expression was evident, which is consistent with previous reports[13,35]. In agreement with our previous study[3], the higher proliferative capacity of CD8^+^ T cells associated with protective alleles was noted. The diminished proliferative capacity of CTLs-restricted by HLA-B*35Px, and lower GzmB and perforin expression in these HIV-epitope specific CD8^+^ T cells may represent a potential mechanism by which HIV-1 avoids CTL control resulting in disease progression in these individuals.

The effectiveness of an effector CTL is governed by external or internal forces, especially, by the regulation imposed by cells that are specialized for this role e.g. Tregs[4]. Our previous studies showed that HLA-B*27/B*57-restricted CTLs evade Tregs mediated suppressing and therefore can eliminate infected cells whereas CTLs-restricted by non-HLA-B*27/B57 become suppressed by Tregs and therefore exhibit impaired effector functions[3]. In the present study, since we did not find any significant difference between the proliferative capability of CTLs restricted by non-HLA-B*27/B*57 and those restricted by HLA-B*35Px alleles, we reasoned that Tregs may suppress CTLs associated with HLA-B*35Px. Surprisingly, we found that this was not the case and of critical importance was the observation that within the same individual, CTLs restricted by non-HLA-B*27/B*57 became suppressed by Tregs but the presence or absence of Tregs did not impact the proliferative capacity or cytokine production ability of HLA-B*35Px-restricted CTLs. This suggests that another potential mechanism may be involved in the impaired effector function of CTLs-restricted by the HLA-B*35Px. Of interest, evasion of Tregs-mediated suppression may explain the association of autoimmunity with HLA-B*27 and HLA-B*57 alleles (e.g. ankylosing spondylitis and psoriasis) [36,37]. Similarly, the HLA-B*35 allele is associated with autoimmune thyroiditis due to CTL dysfunction [38,39]. Thus, our observations provide an important insight into the role of HLA-B*35 allele in disease susceptibility.

In our previous findings, we showed that there were two divergent outcomes for HIV-specific CD8^+^ CTL during HIV-infection: CTLs restricted by non-HLA-B*27/B57 upregulate TIM-3 when they encounter their cognate epitopes and are subsequently suppressed by Tregs; however, CD8^+^ CTL restricted by “protective” HLA alleles (e.g. HLAB*27 and HLA-B*57) upregulate fewer TIM-3, but more GzmB upon recognition of their cognate epitopes[3]. Although this is speculative, these data, suggest that GzmB and TIM-3 expression may be reciprocally linked. For instance, GzmB may cleave a transcription factor that is required for TIM-3 expression. There is precedence for this in that other cathepsins have been shown to play a role in transcriptional regulation[40]. However, when it comes to CD8^+^ T cells restricted by HLA-B*35Px, we found a significantly lower expression of GzmB and lower frequency of TIM-3 expressing CTLs upon recognition of their cognate epitopes versus not-protective alleles (e.g. non-HLA-B*27/B*57). Lower TIM-3 expression on CTLs restricted by HLA-B*35Px may explain a lack of Tregs-mediated suppression via Gal-9: TIM-3[3]. This observation raised additional questions regarding impaired HIV-specific CD8^+^ T cell proliferation in patients with HLA-B*35Px. To address this question, we speculated that chronic HIV infection results in the upregulation of other co-inhibitory receptors and subsequently CTL exhaustion[41]. We have previously shown that NK cells and CTLs upon chronic HIV-1 antigenic stimulation upregulate various co-inhibitory receptors such as PD-1, CTLA-4, TIM-3, 2B4, CD160, Gal-9, CD39, and VISTA [41,42]. However, because of the limited antigen-specific CTLs, we analyzed only the well-studied co-inhibitory receptors, PD-1, CTLA-4, and TIM-3. We observed that antigen-specific CD8^+^ T cells restricted by HLA-B*35Px upregulated substantial levels of PD-1 when they encountered their cognate epitopes. However, blockade of PD-1 with its potential ligands (e.g. PDL-1 and PDL-2) did not improve the proliferative capacity of these CTLs, which is consistent with another report[34]. A previous study indicated that the interaction of P35Px molecule with ILT4 on DCs renders these cells to an impairment phenotype, which may lead to insufficient adaptive immunity in HLA-B*35Px patients[17]. Thus, there is a possibility that DCs and other APCs by the expression of PDL-1, Gal-9 and/or CD80/CD86 and via interaction with their corresponding receptors modulate CTL functions[22]. The higher affinity of CTLA-4 for CD80/CD86 in competition with CD28 can inhibit T cell activation[43]. Using antigen-specific stimulation, we observed that CD8^+^ T cells restricted by HLA-B*35Px upon recognition of their cognate epitopes selectively upregulate significantly higher levels of CTLA-4 compared to CD8^+^ T cells restricted by protective and non-protective alleles. Interestingly, we observed that the blockade of PD-1 or TIM-3 using Pembrolizumab and an anti-TIM-3 antibody did not but Ipilimumab, using physiological concentrations[30], reversed impaired proliferative capacity of CD8^+^ T cells restricted by HLA-B*35Px. These observations suggest that CTLs restricted by different HLA-alleles when encountering their cognate epitopes may signal through a unique pathway that results in the upregulation of different co-inhibitory receptors or upregulation of lytic effector molecules. One major limitation of our study is that we were unable to determine the mechanism underlying the differential expression of co-inhibitory receptors upon cognate epitope recognition of CTLs-restricted by different HLA-alleles. One may suggest that specific features of epitope-specific T cell receptors (TCRs) restricted by different HLA-alleles can impact T cell function. For examples, TCR sequences from protective CTLs may possess unique features enabling them to engage peptide:HLA in conformations that lead to different functional outcomes. To our knowledge, few groups have analyzed TCR sequences from HIV-1 epitope-specific CTLs to determine if there were any unusual TCR features of B27- or B57-restricted CTLs. The majority of this work has focused on a single B57-restricted epitope specificity (KAFSPEVIPMF; KF11) and has shown that KF11-specific CTL possess TCR with enhanced usage of either Vβ7[44] or Vβ19[45]. HLA-B*5701-restricted TCRs were able to recognize multiple variants of the KF11 epitope, which was surmised to account for the limited mutational escape in KF11 in individuals with HLA-B*5701. Another study described limited TCR Vβ usage and unusually long CDR3 regions of TCR from HLA-B08-restricted FLKEKGGL (FL8)-specific CTLs. This group also showed that these TCRs recognize naturally occurring variants of this epitope and are associated with better clinical outcomes[46]. Cross-reactivity of TCR clonotypes has also been documented for CTLs recognizing the B27-restricted KRWIILGLNK (KK10) epitope[47]. Also, it is known that HLA-B*27 is an unusual HLA in that it can form homo-dimers and trimers and can reach the cell surface without being bound by an epitope[48]. Therefore, specific features of the TCRs recognizing different HLAs may explain why some CTLs evade suppression by Tregs or upregulate certain co-inhibitory receptors. Further studies are required to examine these possibilities, and understanding such mechanism(s) would have important clinical implications in other chronic viral infections and cancer. The resistance of CTLs restricted by protective alleles to TIM-3 and CTLA-4 upregulation may be coupled to GzmB expression and other polyfunctionality, representing a qualitative difference. However, upregulation of TIM-3 and CTLA-4 on CTLs restricted by non-HLA-B*27/B*57 and HLA-B*35Px, respectively, may account for an explanation for their impaired functionality. This was also marked at the gene level by a higher expression of Eomes in antigen-specific CTLs restricted by HLA-B*35Px. Eomes is reported to be directly associated with an exhausted CD8^+^ T cell phenotype [49], which can explain the limited proliferative capacity of HLA-B*35Px restricted CTLs. However, the lower expression of T-bet in CD8^+^ T cells restricted by HLA-B*35Px suggests a differential link for this transcription factor in CTL exhaustion. This hypothesis is supported by the fact that poor upregulation of T-bet results in impaired cytotoxic granules and the inability to kill virus-infected cells in HIV-infected chronic progressors[50]. These observations support the concept that the exhausted CTL phenotype in CD8^+^ T cells restricted by HLA-B*35Px might be related to an inverse expression between T-bet and Eomes[50]. These results may aid to explain impaired functionality of HLA-B*35Px restricted CTLs even at the gene level (e.g. lower IL-2, IFN-γ, and TNF-α), which is consistent with an exhausted T cell phenotype[51]. Of note, higher expression of blimp-1/Prdm1 mRNA in HIV-progressors versus LTNPs has been reported which agrees with our observations[52].

Although our report, provides a novel insight into the impact of HLA-B*35Px on the expression of co-inhibitory receptors on antigen-specific CTLs, we are aware of our study limitations. For example, our studies were performed on a small cohort and thus we are hesitant for the general implication of our findings. However, recruiting additional HIV+ HLA-B*35Px in the era of ART treatment is almost impossible. The other limitation is that we were unable to analyze the co-expression of other co-inhibitory receptors due to cell and/or tetramer dye limitations.

Currently, anti-CTLA-4 antibodies such as ipilimumab have shown to provide a survival benefit in cancer patients[53,54]. It is well apparent that immunotherapy with immune checkpoint blockers (e.g. ipilimumab or pembrolizumab) can expand activated antigen-specific CTLs[55]. Therefore, the clinical efficacy of such therapeutic approaches to reinvigorate exhausted CTLs in HIV-1 infection may be considered.

In summary, we here show that CD8^+^ T cells restricted by HLA-B*27/B57 evade Tregs mediated suppression whereas CD8^+^ T cells restricted by HLA-B*35Px exhibit an impaired phenotype regardless of Tregs presence. Besides, CD8^+^ T cells restricted by HLA-B*35Px unlike non-HLA-B*27/B57 do not upregulate substantial levels of TIM-3 when encountering their cognate epitopes but instead upregulate CTLA-4. The sustained expression of Eomes, blimp-1 and poor expression of T-bet suggest a dysfunctional phenotype for CTLs restricted by HLA-B*35Px, which fail to elicit an efficient response to control viral replication and thus are associated with disease progression. Future studies aim to understand how HLA-restriction impacts the expression of co-inhibitory receptors may provide important information for strategies to modulate their expression by targeting epigenetic transcription to alter the immune response. Overall, this study provides a novel potential mechanism underling the detrimental role of HLA-B*35Px in HIV pathogenesis.

## Material and methods

### Study group

10 Long-term non-progressors (LTNPs) and 5 Rapid-progresses (PRs) were subjected to our studies. HIV-1 patients were recruited from the Northern Alberta HIV Cohort. Clinical data and HLA genotypes of the patients are shown in S1 Table. In addition, some frozen PBMCs were obtained from the Centre for AIDS Research (CFAR)-University of Washington, Seattle.

### Ethics statement

This study was approved by the institutional research review boards at the University of Alberta and written informed consent was obtained from all the participants in the study protocol numbers Pro000046064 and Pro000070528.

### Cell isolation and *in vitro* assays

The peripheral blood mononuclear cells (PBMCs) were isolated from fresh blood or through leukapheresis process at the University of Alberta Hospital (protocol # Pro000046064). Samples were subjected to Ficoll-Paque gradients and cultured in RPMI 1640 (Sigma) supplemented with 10% fetal bovine serum (Sigma) and 1% penicillin/streptomycin (Sigma). In most studies, frozen PBMCs were thawed and used. Tregs were isolated from PBMCs according to the manufacturing instruction (STEMCELL Technologies) and our previous reports [3,56]. Proliferation assay was performed using CFSE dye according to our previous protocols[3, 27]. Surface staining and intracytoplasmic cytokine staining (ICS) were performed as we have reported elsewhere [3,56,57]. Briefly, PBMCs were cultured at 1x10^6^ cells/well in RMPI culture media supplemented with 10% FBS and antibiotics. PBMCs were stimulated with their corresponding epitopes, one epitope/well (2 μg/ml), and incubated at 37^ο^ C for 66 h then Golgi blocker was added for the remaining 6hr before staining. In some experiments, anti-PD-1 (Pembrolizumab 5 μg/ml), anti-CTLA-4 (Ipilimumab 5 μg/ml) or anti-TIM-3 (10 μg/ml LEAF anti-Tim-3 (clone F38-2E2, BioLegend) antibodies were used as we have reported elsewhere[29,30,58].

### Epitope mapping and ELISpot assay

The mapping of HIV epitope specificities was performed in each patient as described previously(3). S2 and S3 Tables show the HLA-type and epitope-specific responses for each patient. LTNPs or PRs were mapped, using potential T cells epitopes (PTE) provided by NIH AIDS Research and Reference Reagent Program (Bethesda, MD). Based on recognized PTE responses and Los Alamos HIV sequence database, we identified potential epitopes. Potential epitopes were synthesized (Sigma Aldrich) with purity above 90% and then tested by performing ELISpot. In brief, we cultured 2 X 10^5^ cells per well and stimulated with 2 μg ml^–1^ of their peptides/cognate epitopes. Positive responses were designated when the number of spot-forming cells was twice background and at least 50 spot-forming cells/10^6^ PBMC, as we have described elsewhere[3].

### Flow cytometry

Fluorophore antibodies and cytokines were purchased from BD Biosciences, Thermo Fisher Scientific, Biolegend, and R&D. We used the following antibodies in our study: anti-CD3 (SK7), anti-CD4 (RPA-T4), anti-CD8 (RPA-T8), anti-PD-1 (MIH4), anti-TIM-3(7D3), anti-CTLA-4 (BN13), anti-CD25 (M-A251), anti-FOXP3 (150D/E4), anti-CD137 (4B4-1), anti-CD127 (HIL-7R-M21), anti-Perforin (δG9), anti-Granzyme B (GB11), anti-IL-2 (MQ1-17H12), anti-TNF-α (MAB11), and IFN-γ (4S.B3). Dextramers were obtained from IMMUDEX. To evaluate cell viability, LIVE/DEAD Kit (Thermo Fisher Scientific) was used. After fixation with Paraformaldehyde (PFA 4%), stained cells were acquired on an LSRII flow cytometer (BD Biosciences) and analyzed using FlowJo Version 7.2.2 software.

### Gene expression analysis

RNA isolation and qPCR were performed as previously described [27,59]. TaqMan qPCR method (Applied Biosystems) was performed using 5 ng/μl of cDNA as a template. We used the following gene expression probe assays: Eomes (QT00026495), TNF-α (QT00029162), IFN-γ (QT00000525), T-bet (QT00042217), Blimp-1(Hs00153357_m1) and IL-2 (QT00015435). We ran each sample in duplicate on Real-Time PCR Detection System (Applied Biosystem). GAPDH (glyceraldehyde-3-phosphate dehydrogenase) sense primer 5′-CCACCCATGGCAAATTCCATGGCA-3′ was used as a reference gene, and the gene expression of the targeted genes was calculated by the 2^-ΔΔCt^ method.

### Statistical analyses

A comparison between the two groups was performed by the non-parametric Mann-Whitney test. We used one-way ANOVA to compare more than two groups. Prism analysis software was used for statistical analysis. The results are displayed as mean ± SEM and *p*-value less than 0.05 was considered as statistically significant.

## Supporting information

S1 Table Showing patients clinical information including their HLA-type, disease status (e.g. Long-term nonprogressors (LTNPs) or Progressors (PR)), sex, viral load, and CD4 T cell count. (DOCX)

S2 Table Presenting the sequence of all the recognized epitopes by each HIV-infected individual defined as “progressors” including their HLA-allele restriction. Also, this table shows the number of IFN-γ secreting cells in response to each corresponding epitope as measured by ELISpot assay.
(DOC)

S3 Table Presenting the sequence of all the recognized epitopes by each HIV-infected individual defined as “Long-term nonprogressors” including their HLA-allele restriction. Also, this table shows the number of IFN-γ secreting cells in response to each corresponding epitope as measured by ELISpot assay.
(DOC)

S1 Fig **(A)** Cumulative data showing percentages of IL-2 secreting CD8^+^ T cells restricted by non-HLA-B*27/B*57 and HLA-B*35 in HIV-infected individuals having HLA-B*35Px following stimulation with their cognate epitopes (2 μg/ml) for 72 hrs as measured by ICS. (**B)** Cumulative data showing percentages of IFN-γ secreting CD8^+^ T cells restricted non-HLA-B*27/B*57 and HLA-B*35 in HIV-infected individuals having HLA-B35Px following stimulation of PBMCs with their cognate epitopes (2 μg/ml) for 72 hrs using ICS. (**C**) Cumulative data showing percentages of TNF-α secreting CD8^+^ T cells restricted by non-HLA-B*27/B*57 and HLA-B*35 in HIV-infected individuals having HLA-B35Px following stimulation with their cognate epitopes (2 μg/ml) for 72 hrs as measured by ICS. Each point represents data from an epitope.
(TIFF)
