# Supplementary material for: Selective Upregulation of CTLA-4 on CD8+ T Cells Restricted by HLA-B*35Px Renders them to an Exhausted Phenotype in HIV-1 infection
Source: PLoS Pathog. 2020 Aug 6;16(8):e1008696. doi: 10.1371/journal.ppat.1008696 (PMC7410205; doi:10.1371/journal.ppat.1008696)
Supplement: S1 Table — (DOCX) [file ppat.1008696.s001.docx]

**Table 1** Participants clinical data and HLA^1^ genotypes.

| PTID^2^ | HLA-A | | HLA-B | | HLA-C | | LTNP^3^ or PR | Sex | Plasma  Viral load | CD4 Count |
| --- | --- | --- | --- | --- | --- | --- | --- | --- | --- | --- |
| LNP-01 | 02G1 | 24G1 | 2705 | 1501 | 0102 | 0304 | LTNP | M | 250 | 623 |
| LNP-02 | 0101 | 2601 | 4901 | 5701 | 0602 | 07G1 | LTNP | M | <50 | 581 |
| LNP-03 | 03G1 | 3101 | 2705 | 38 | 0202 | 1801 | LTNP | M | <50 | 503 |
| LNP-04 | 03G1 | 68G1 | 5301 | 5703 | 04G1 | 1801 | LTNP | F | 760 | 444 |
| LNP-05 | 0301 | 3101 | 5701 | 2705 | 0302 | 0602 | LTNP | M | <50 | 788 |
| LNP-06 | 0201 | 0101 | 5701 | 4001 | 0304 | 0602 | LTNP | M | <50 | 855 |
| LNP-07 | 24G1 | 2501 | 3901 | 5701 | 0602 | 1203 | LTNP | M | 125 | 783 |
| LNP-08 | 0101 | 0301 | 2705 | 5701 | 0202 | 0602 | LTNP | M | <50 | 801 |
| LNP-09 | 24G1 | 3101 | 2702 | 2705 | 0202 | 0202 | LTNP | F | <50 | 796 |
| LNP-10 | 0101 | 0201 | 5701 | 1501 | 0304 | 0602 | LTNP | M | <50 | 502 |
| PR01 | 0101 | 0301 | 0801 | 3503 | 0401 | 0701 | PR | F | 385,000 | 239 |
| PR02 | 24G1 | 24G1 | 3502 | 3801 | 04G1 | 1203 | PR | F | 611,600 | 327 |
| PR03 | 03G1 | 1101 | 3503 | 5301 | 04G1 | 04G1 | PR | M | 154,000 | 312 |
| PR04 | 0101 | 02G1 | 0801 | 3503 | 04G1 | 07G1 | PR | M | 942,000 | 233 |
| PR05 | 03G1 | 1101 | 3503 | 44G1 | 04G1 | 0501 | PR | M | 65,700 | 271 |

^1^Human Leukocyte antigen

^2^Patient identification number

^3^LTNP (Long term non-progressor) or PR (progressor)
