# Supplementary material for: Selective Upregulation of CTLA-4 on CD8+ T Cells Restricted by HLA-B*35Px Renders them to an Exhausted Phenotype in HIV-1 infection
Source: PLoS Pathog. 2020 Aug 6;16(8):e1008696. doi: 10.1371/journal.ppat.1008696 (PMC7410205; doi:10.1371/journal.ppat.1008696)
Supplement: S2 Table — Also, this table shows the number of IFN-γ secreting cells in response to each corresponding epitope as measured by ELISpot assay. (DOC) [file ppat.1008696.s002.doc]

**Table 2** Measured epitope-specific CD8^+^ T cells IFN-γ response.

| **PTID^1^** | | **HLA^2^ Allele** | | **Epitope Sequence** |  | **ELISpot (IFN-γ)** |  | |  |  |  | |
| --- | --- | --- | --- | --- | --- | --- | --- | --- | --- | --- | --- | --- |
| PR01 | | B3503 | | [FPVKPQVPL](http://www.hiv.lanl.gov/content/immunology/ctl_search?results=Search;id=54548) |  | 1874 |  | |  |  |  | |
| PR01 | | A08 | | FLKEKGGL |  | 254 |  | |  |  |  | |
| PR01 | | B08/35 | | NPDCKTIL |  | 202 |  | |  |  |  | |
| PR01 | | B3503 | | HPVHAGPI |  | 127 |  | |  |  |  | |
| PR01 | | A01 | | RRGWEVLKY |  | 2475 |  | |  |  |  | |
| PR01 | | B08 | | RQGLERALL |  | 135 |  | |  |  |  | |
| PR02 | | B38 | | YSTQVDPDL |  | 725 |  | |  |  |  | |
| PR02 | | A24 | | RYPLTFGWCF |  | 895 |  | |  |  |  | |
| PR02 | | B3502 | | DPNPQEVVL |  | 460 |  | |  |  |  | |
| PR02 | | B38 | | VWKDAETTL |  | 457 |  | |  |  |  | |
| PR02 | | B38 | | EIWDNMTWL |  | 147 |  | |  |  |  | |
| PR03 | | B3503 | | DIWDNMTWM |  | 515 |  | |  |  |  | |
| PR03 | | A03 | | QVPLRPMTYK |  | 285 |  | |  |  |  | |
| PR03 | | A03 | | AVDLSHFLK |  | 463 |  | |  |  |  | |
| PR03 | | B53 | | YPLTFGWCF |  | 168 |  | |  |  |  | |
| PR03 | | B53 | | DPEKEVLVW |  | 365 |  | |  |  |  | |
| PR03 | | A03 | | RLRPGGKKK |  | 487 |  | |  |  |  | |
| PR03 | | A11 | | ATLYCVHQR |  | 1147 |  | |  |  |  | |
| PR03 | | B3503 | | NPDCKTIL |  | 86 |  | |  |  |  | |
| PR03 | | B53 | | QASQDVKNW |  | 1182 |  | |  |  |  | |
| PR03 | | A03 | | QIYAGIKVK |  | 243 |  | |  |  |  | |
| PR04 | | A02 | | GLADQLIHL |  | 2046 |  | |  |  |  | |
| PR04 | | A2 | | ALAALITPK |  | 928 |  | |  |  |  | |
| PR04 | | B08 | | FLKEKGGL |  | 3180 |  | |  |  |  | |
| PR04 | | A02 | | SLYNTVATL |  | 194 |  | |  |  |  | |
| PR04 | | B08 | | ELRSLYNTV |  | 626 |  | |  |  |  | |
| PR04 | | B08 | | EIYKRWII |  | 183 |  | |  |  |  | |
| PR04 | | B3503 | | QVTNSATIM |  | 75 |  | |  |  |  | |
| PR04 | | B3503 | | TVLDVGDAY |  | 1128 |  | |  |  |  | |
| PR04 | | B08 | | RGRQKVVSL |  | 110 |  | |  |  |  | |
| PR04 | | B3503 | | DPNPQEVVL |  | 2346 |  | |  |  |  | |
| PR04 | | A02 | | RQGFERALL |  | 1422 |  | |  |  |  | |
| PR04 | | A02 | | LLNTTAIVV |  | 361 |  | |  |  |  | |
| PR04 | | B3503 | | TAVPWNASW |  | 2300 |  | |  |  |  | |
| PR05 | | A03 | | AVNLSHFLK |  | 395 |  | |  |  |  | |
| PR05 | | B3503 | | FPVRPQVPL |  | 277 |  | |  |  |  | |
| PR05 | | B3503 | | FPDWQNYTP |  | 92 |  | |  |  |  | |
| PR05 | | A301 | | RLRPGGKKK |  | 262 |  | |  |  |  | |
| PR05 | | B44 | | AEQASQDVKNW |  | 425 |  | |  |  |  | |
| PR05 | | B3503 | | HPVHAGPV |  | 967 |  | |  |  |  | |
| PR05 | | B3503 | | QVTNSATIM |  | 117 |  | |  |  |  | |
| PR05 | | A03 | | NSATIMMQR |  | 247 |  | |  |  |  | |
| PR05 | A11 | | QIYAGIKVK 475 | | | | |  | | | |  |
| PR05 | | B3503 | | VPLTAEAEM |  | 425 |  | |  |  |  | |
| PR05 | | A11 | | QIIEQLIKK |  | 177 |  | |  |  |  | |
| PR05 | | B44 | | EEHEKYHSNW |  | 82 |  | |  |  |  | |
| PR05 | | B3503 | | DPNPQEVVL |  | 130 |  | |  |  |  | |

^1^Patient Identification Number ^2^Human Leukocyte Antigen
