# Supplementary material for: Selective Upregulation of CTLA-4 on CD8+ T Cells Restricted by HLA-B*35Px Renders them to an Exhausted Phenotype in HIV-1 infection
Source: PLoS Pathog. 2020 Aug 6;16(8):e1008696. doi: 10.1371/journal.ppat.1008696 (PMC7410205; doi:10.1371/journal.ppat.1008696)
Supplement: S3 Table — Also, this table shows the number of IFN-γ secreting cells in response to each corresponding epitope as measured by ELISpot assay. (DOC) [file ppat.1008696.s003.doc]

**Table 3** Measured epitope-specific CD8^+^ T cells IFN-γ response.

| **PTID^1^** | **HLA^2^ Allele** | **Epitope Sequence** |  | **ELISpot (IFN-γ)** |  |  |  |
| --- | --- | --- | --- | --- | --- | --- | --- |
| LNP01 | A02 | TAFTIPSI |  | 187 |  |  |  |
| LNP01 | A02 | SLYNTVATL |  | 333 |  |  |  |
| LNP01 | A24 | RYLKDQQLL |  | 188 |  |  |  |
| LNP01 | B27 | GLNKIVRMY |  | 1102 |  |  |  |
| LNP01 | B27 | IRLRPGGKK |  | 199 |  |  |  |
| LNP01 | B27 | KRWIILGLNK |  | 1623 |  |  |  |
| LNP02 | B57 | KAFSPEVIPMF |  | 2018 |  |  |  |
| LNP02 | B57 | YFPDWQNYT |  | 1120 |  |  |  |
| LNP02 | B57 | ISPRTLNAW |  | 950 |  |  |  |
| LNP02 | B57 | IVLPEKDSW |  | 234 |  |  |  |
| LNP02 | A26 | EVIPMFSAL |  | 1918 |  |  |  |
| LNP03 | A03 | RLRPGGKKK |  | 193 |  |  |  |
| LNP03 | B27 | IRLRPGGKK |  | 744 |  |  |  |
| LNP03 | B27 | KRWIILGLNK |  | 1056 |  |  |  |
| LNP04 | A03 | AIFQSSMTK |  | 105 |  |  |  |
| LNP04 | A03 | QVPLRPMTYK |  | 211 |  |  |  |
| LNP04 | A03 | IRLRPGGKKK |  | 687 |  |  |  |
| LNP04 | B53 | QASQEVKNW |  | 501 |  |  |  |
| LNP04 | B57 | ISPRTLNAW |  | 622 |  |  |  |
| LNP04 | B57 | KAFSPEVIPMF |  | 1901 |  |  |  |
| LNP04 | B57 | IVLPEKDSW |  | 423 |  |  |  |
| LNP05 | A03 | RLRDLLLIVTR |  | 189 |  |  |  |
| LNP05 | B57 | IVLPEKDSW |  | 681 |  |  |  |
| LNP05 | B57 | QASQEVKNW |  | 543 |  |  |  |
| LNP05 | A03 | RLRPGGKKK |  | 321 |  |  |  |
| LNP05 | A03 | RLRPGGKKKY |  | 156 |  |  |  |
| LNP05 | B57 | KAFSPEVIPMF |  | 1724 |  |  |  |
| LNP06 | B57 | KTAVQMAVF |  | 960 |  |  |  |
| LNP06 | B57 | KAFSPEVIPMF |  | 345 |  |  |  |
| LNP06 | B40 | IEIKDTKEAL |  | 211 |  |  |  |
| LNP06 | A02 | ILKEPVHGV |  | 679 |  |  |  |
| LNP06 | B57 | YFPDWQNYT |  | 555 |  |  |  |
| LNP06 | B57 | ISPRTLNAW |  | 1102 |  |  |  |
| LNP07 | B57 | AVRHFPRIW |  | 148 |  |  |  |
| LNP07 | B57 | QASQEVKNW |  | 1890 |  |  |  |
| LNP07 | B57 | ISPRTLNAW |  | 1840 |  |  |  |
| LNP07 | B57 | KAFSPEVIPMF |  | 472 |  |  |  |
| LNP07 | B57 | TSTLQEQIGW |  | 315 |  |  |  |
| LNP08 | A03 | RLRPGGKKK |  | 607 |  |  |  |
| LNP08 | A03 | MVHQAISPR |  | 66 |  |  |  |
| LNP08 | A03 | LVWASRELERF |  | 72 |  |  |  |
| LNP08 | A03 | KIRLRPGGK |  | 1112 |  |  |  |
| LNP08 | B57 | ISPRTLNAW |  | 2495 |  |  |  |
| LNP08 | B57 | KAFSPEVIPMF |  | 2876 |  |  |  |
| LNP08 | B57 | TSTLQEQIGW |  | 777 |  |  |  |
| LNP08 | B57 | IVLPEKDSW |  | 1115 |  |  |  |
| LNP09 | A24 | RYPLTFGWCF |  | 298 |  |  |  |
| LNP09 | B27 | RRGWEALKY |  | 142 |  |  |  |
| LNP09 | B27 | VRYPLTFGW |  | 501 |  |  |  |
| LNP10 | B57 | ISPRTLNAW |  | 2231 |  |  |  |
| LNP10 | B57 | KAFSPEVIPMF |  | 2122 |  |  |  |
| LNP10 | B57 | IVLPEKDSW |  | 1781 |  |  |  |
| LNP10 | A02 | FLGKIWPSYK |  | 321 |  |  |  |
| LNP10 | A02 | [PIVLPEKDSW](http://www.hiv.lanl.gov/content/immunology/ctl_search?results=Search;id=485;id=486) |  | 602 |  |  |  |

^1^ Patient Identification Number

^2^ Human Leukocyte Antigen
