# Supplementary figures and images for: Selective Upregulation of CTLA-4 on CD8+ T Cells Restricted by HLA-B*35Px Renders them to an Exhausted Phenotype in HIV-1 infection
Source: PLoS Pathog. 2020 Aug 6;16(8):e1008696. doi: 10.1371/journal.ppat.1008696 (PMC7410205; doi:10.1371/journal.ppat.1008696)

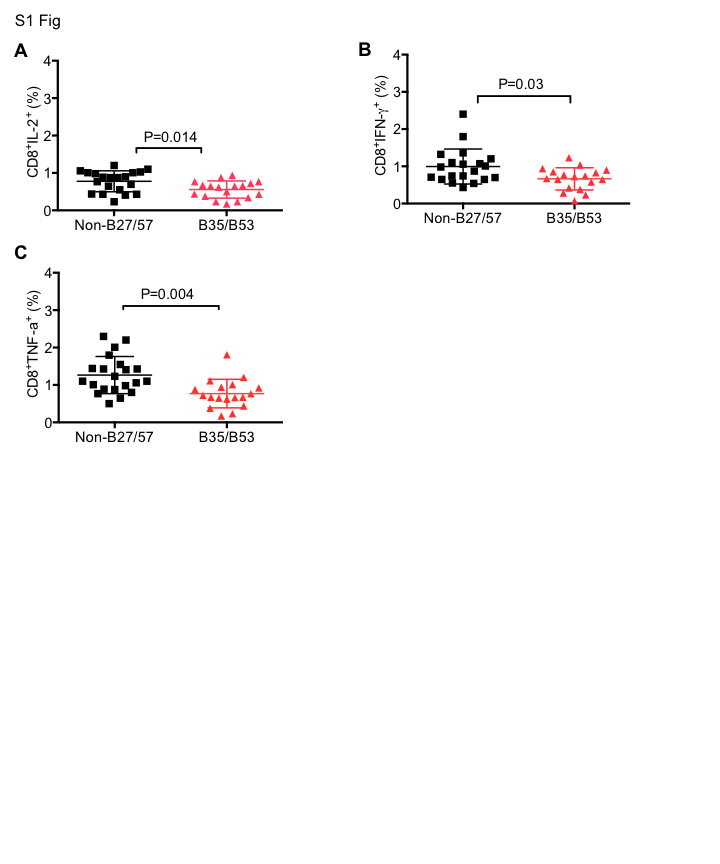

Supplement: S1 Fig — (A) Cumulative data showing percentages of IL-2 secreting CD8+ T cells restricted by non-HLA-B*27/B*57 and HLA-B*35 in HIV-infected individuals having HLA-B*35Px following stimulation with their cognate epitopes (2 μg/ml) for 72 hrs as measured by ICS. (B) Cumulative data showing percentages of IFN-γ secreting CD8+ T cells restricted non-HLA-B*27/B*57 and HLA-B*35 in HIV-infected individuals having HLA-B35Px following stimulation of PBMCs with their cognate epitopes (2 μg/ml) for 72 hrs using ICS. (C) Cumulative data showing percentages of TNF-α secreting CD8+ T cells restricted by non-HLA-B*27/B*57 and HLA-B*35 in HIV-infected individuals having HLA-B35Px following stimulation with their cognate epitopes (2 μg/ml) for 72 hrs as measured by ICS. Each point represents data from an epitope. (TIFF) [file ppat.1008696.s004.tiff]
